# Unravelling the role of key amino acid residues of the parainfluenza fusion peptide in membrane fusion

**DOI:** 10.1039/d5cb00058k

**Published:** 2025-05-21

**Authors:** Mariana Valério, Carolina C. Buga, Diogo A. Mendonça, Miguel A. R. B. Castanho, Manuel N. Melo, Cláudio M. Soares, Diana Lousa, Ana Salomé Veiga

**Affiliations:** a Instituto de Tecnologia Química e Biológica, Universidade Nova de Lisboa Av. da República 2780-157 Oeiras Portugal claudio@itqb.unl.pt dlousa@itqb.unl.pt; b Gulbenkian Institute for Molecular Medicine Av. Professor Egas Moniz 1649-028 Lisboa Portugal; c Faculdade de Medicina, Universidade de Lisboa Av. Professor Egas Moniz 1649-028 Lisboa Portugal aveiga@medicina.ulisboa.pt

## Abstract

Parainfluenza viruses enter host cells by fusing their envelope with the cell membrane. In this process mediated by the fusion glycoprotein, the fusion peptide plays an essential role in membrane binding and triggering fusion. Previously, we demonstrated that the parainfluenza fusion peptide (PIFP) oligomerizes into porelike structures within the membrane, leading to membrane perturbations, fusion, and leakage. Additionally, we identified two key amino acid residues in the PIFP, F103 and Q120, which are important in inducing lipid tail protrusion and maintaining peptide–peptide interactions, respectively. Here, we seek to elucidate the role of these two residues in the PIFP function by studying the impact of F103A and Q120A substitutions on peptide activity. We compared the substituted peptides with the native peptide using biophysical experiments and molecular dynamics (MD) simulations. Our results show that the F103A substitution significantly impairs PIFP's interaction with the membrane and its ability to induce lipid mixing and membrane leakage in experimental assays. Moreover, a decrease in lipid perturbation and water flux through the membrane was observed in the MD simulations. In contrast, the Q120A substitution appears to have minimal impact on membrane interaction and PIFP-induced membrane leakage. Interestingly, a pronounced change in the interpeptide interactions within the membrane of the substituted peptides was observed in the MD simulations. These findings provide crucial insights into the potential role of F103 and Q120 in PIFP activity: the N-terminal phenylalanine (F103) is pivotal for membrane insertion and fusion, while the Q120 is crucial for regulating peptide oligomerization and pore formation.

## Abstract 2

Parainfluenza viruses entry relies on membrane fusion promoted by the fusion peptide - PIFP. F103A and Q120A substitutions impair the PIFP fusogenic properties, revealing key amino acid residues for its function.

## Graphical abstract

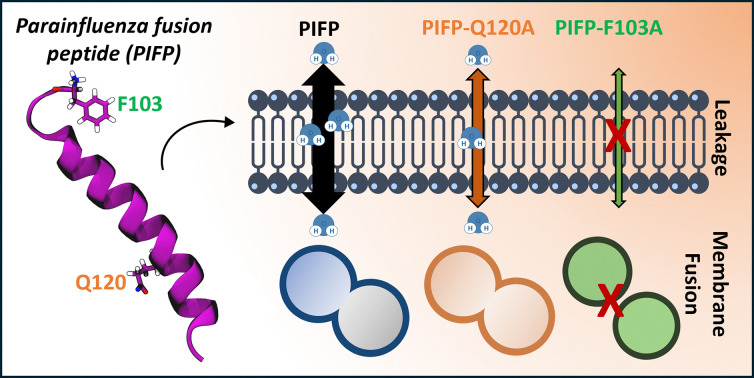

## Introduction

The Paramyxoviridae family includes some of the most critical pathogens in human and animal health, due to their capacity to cause widespread outbreaks and severe illness.^1^ This family of enveloped, single-stranded RNA viruses includes the Hendra, Nipah, and parainfluenza viruses (PIVs).^1^ Although PIVs are responsible for a considerable global disease burden, particularly among children,^2^ there are no approved antiviral treatments or vaccines available to effectively mitigate PIVs-induced diseases.^3^ Therefore, it is of utmost importance to better understand the PIVs entry process into host cells to explore new strategies for preventing or treating these viral infections.

**Fig. 1:**
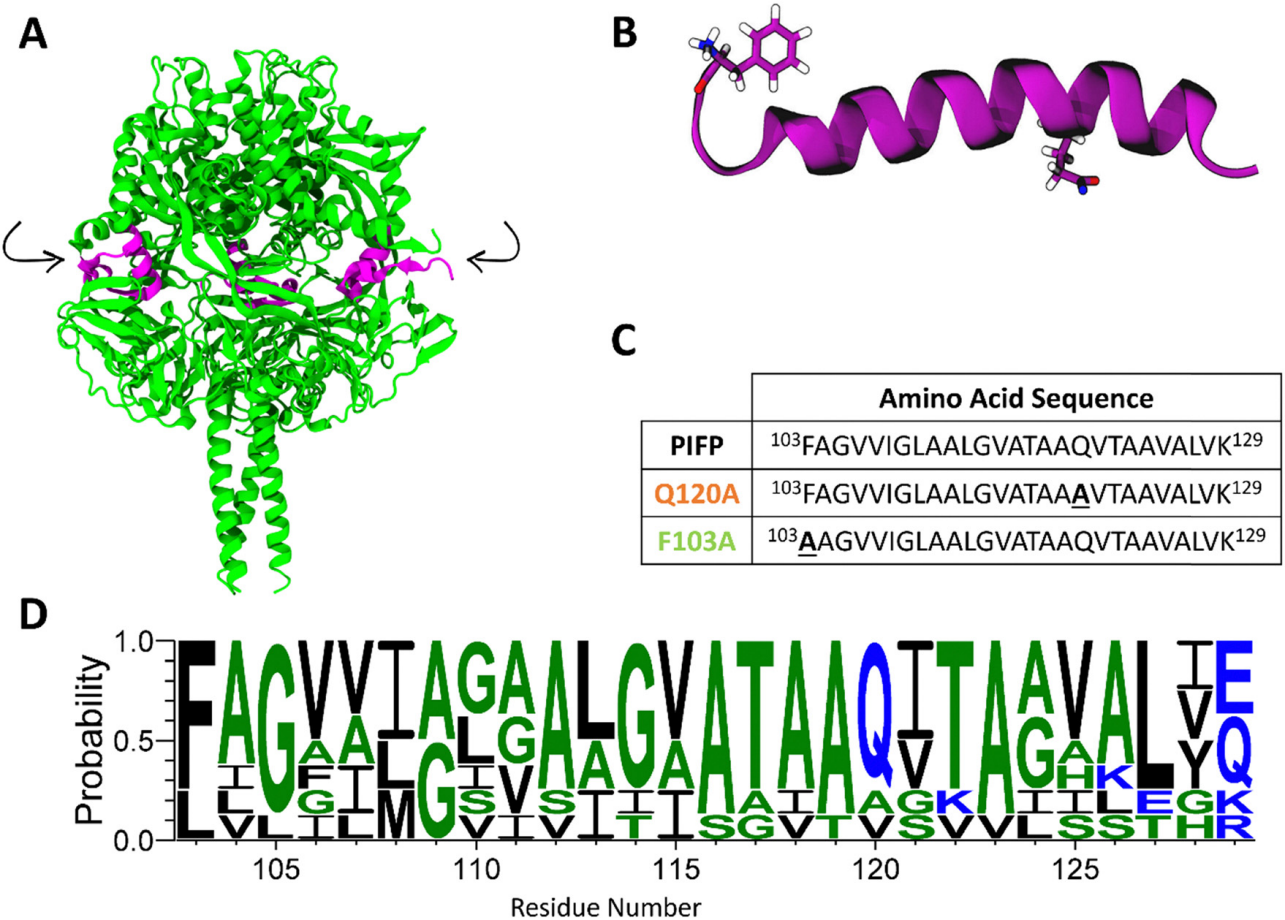
Parainfluenza fusion peptide. (A) Cartoon representation of the crystal structure of the cleaved prefusion F protein trimer, 4GIP, viewed from the side, with the FP highlighted in pink. Arrows indicate the protease cleavage sites. After cleavage, the F protein adopts its active form, F_1_, in which the F103 becomes the N-terminal residue of both the PIFP and the F_1_ protein. (B) Molecular representation of the PIFP secondary structure when inserted in the hydrophobic environment of a membrane. The backbone is shown in purple and the side-chains of the F103 and Q120 residues are shown in sticks. (C) Amino acid sequences for the PIFP and substituted peptides (PIFP–Q120A and PIFP–F103A). Substituted residues shown in bold and underlined. (D) Sequence conservation of the fusion peptide across diverse human and zoonotic paramyxoviruses. Colour is used to show hydrophobicity of the residues (blue: hydrophilic, green: neutral, and black: hydrophobic). Sequence logo generated with WebLogo.^9^

PIVs rely on the fusion between their envelope and the host plasma membrane to insert the viral genome into the host cell. The fusion process is orchestrated by the coordinated action of the receptor-binding protein hemagglutinin-neuraminidase (HN) and the fusion glycoprotein (F protein). The F protein, initially synthesized as a precursor (F_0_), is cleaved in the process of virion assembly to form a pre-fusion F_1_ trimer where the hydrophobic fusion peptide (FP) region is initially concealed (Fig. 1A). In the first step of PIVs life cycle, HN binds to sialic acid-containing receptor molecules on the host cell, which triggers F protein structural changes. This exposes the FP, enabling its insertion into the host membrane. Subsequently, the F protein refolds into a stable post-fusion structure, driving viral envelope and cell membrane fusion. The viral genetic material is then released into the target cell.^4–8^

In a previous study, we have used parainfluenza virus 5 (PIV5) as a model to elucidate the mechanism by which the parainfluenza fusion peptide (PIFP) (Fig. 1B) mediates the membrane fusion process. Since functional regions like the PIFP are generally conserved across viral families, studying the FP from PIV5 can be useful to a broader understanding of PIFP-mediated processes.^10^ We showed that, when present at high concentrations in an anionic membrane, the PIFP can promote fusion and/or membrane leakage through the formation of a water-permeable porelike structure. This structure promotes lipid head intrusion and lipid tail protrusion, key steps in the fusion process.^11^ Rather than merely disrupting membrane packing, fusion peptides are increasingly recognized as active drivers of the fusion process through the controlled formation of pores, which create a favourable environment for membrane fusion. Similar mechanisms have been described for other viral fusion peptides, such as that of influenza, where stable FP-induced pores increase membrane permeability and facilitate the merging of lipid bilayers.^12,13^ Moreover, we have pinpointed two amino acid residues, F103 and Q120, that might play key roles in the PIFP activity. The numbering for F103 and Q120 corresponds to the amino acid residue position in the F_0_ protein sequence before cleavage into the F_1_ active form. Upon cleavage, residue F103 becomes the N-terminus of the PIFP and of the F_1_ protein (as shown in Fig. 1). It is therefore more exposed to the solvent and available for membrane interaction. Additionally, these residues are highly conserved across diverse human and zoonotic paramyxoviruses—including PIV5, Hendra, Nipah, measles, mumps, Newcastle disease virus, respiratory syncytial virus, and human metapneumovirus (Fig. 1D). This suggests that F103 and Q120 play essential roles in maintaining the structural integrity and/or functional activity of the F protein across the paramyxovirus family, reinforcing the broader relevance of these findings beyond PIV5.

In our previous work, MD simulations showed that F103 interacts with lipid head-groups leading to lipid head intrusion and lipid tail protrusion, which are features that have been shown to precede and facilitate lipid mixing events.^11–14^ On the other hand, the Q120 amino acid residue was shown to establish stable peptide–peptide interactions between the peptide monomers that form the porelike structure. In addition, work developed by Donald *et al.* showed that this residue is involved in water interactions in the interior of the porelike structure.^14^ Building on these findings, the present study aims to elucidate the role of F103 and Q120 in the PIFP oligomerization and its ability to induce membrane fusion. We focus on two substituted versions of the PIFP, where either F103 or Q120 are replaced by alanine (PIFP–F103A and PIFP–Q120A, respectively). We conducted a comprehensive analysis of the specific roles of F103 and Q120 in PIFP activity, integrating experimental biophysical data with results from coarse-grain (CG) and all-atom (AA) MD simulations. The results revealed the importance of the N-terminal phenylalanine (F103) in both lipid mixing and membrane leakage induced by the peptide. In contrast, Q120 had a more limited impact on membrane leakage and no significant effect on lipid mixing, but did alter the number and type of peptide–peptide interactions formed within the membrane. Overall, this study enhances the understanding of the consequences of these specific substitutions on PIFP function within the context of the host membrane, shedding light on the distinct roles of these amino acid residues in membrane fusion.

## Methods

### Peptide synthesis

PIFP, PIFP–F103A and PIFP–Q120A were synthesized by Bachem AG (Bubendorf, Switzerland) using solid phase peptide synthesis with a purity >90%. The three peptides comprise the residues 103–129 of the PIV5 fusion protein (Fig. 1C). However, the PIFP–F103A has a substitution of the phenylalanine 103 to an alanine, and PIFP–Q120A has a substitution of the glutamine 120 to an alanine. Each peptide was modified with 8-amino-3,6-dioxaoctanoic acid bridging a poly-lysine tail at the C-terminus to enhance stability and solubility. No modifications were made at the N-terminus.

### Chemicals and reagents

1-Palmitoyl-2-oleoyl-*sn-glycero*-3-phosphocholine (POPC), 1-palmitoyl-2-oleoyl-*sn-glycero*-3-phospho-l-serine (POPS), 1,2-dioleoyl-*sn-glycero*-3-phosphoethanolamine-*N*-(7-nitro-2-1,3-benzoxadiazol-4-yl) (NBD-PE), and 1,2-dioleoyl-*sn-glycero*-3-phosphoethanolamine-*N*-(lissamine rhodamine B sulfonyl) (Rhod-PE) were purchased from Avanti Polar Lipids (USA). 5(6)-Carboxyfluorescein (5,6-CF), sodium 2-(4-(2-hydroxyethyl)piperazin-1-yl)ethanesulfonate, 4-(2-hydroxyethyl)piperazine-1-ethanesulfonic acid sodium salt, *N*-(2-hydroxyethyl)piperazine-*N*′-(2-ethanesulfonic acid) (HEPES), sodium chloride (NaCl), sodium hydroxide (NaOH), 3-[(3-cholamidopropyl)dimethylammonio]-1-propanesulfonate hydrate, 3-[(3-cholamidopropyl)dimethyl-ammonio]-1-propane sulfonate, 3-[(3-cholamidopropyl)dimethyl-ammonio]-1-propane sulfonate, zwitterionic, [cholamidopropyl-dimethyl-ammonio]-1-propane sulfonate, 3-3-zwitterionic, [cholamidopropyl-dimethyl-ammonio]-1-propane sulfonate, 3-3-(CHAPS), sodium dodecyl sulfate (SDS), and *t*-octylphenoxypolyethoxyethanol, polyethylene glycol *tert*-octylphenyl ether (Triton X-100) were purchased from Merck (Germany).

### Instrumentation

Liposomes were extruded through nucleopore poly-carbonate membranes (Whatman/Cytiva, UK) using a LiposoFast-Basic plus Stabilizer setup from Avestin (Germany) with Hamilton (Switzerland) syringes. Dynamic light scattering measurements were carried out in a Malvern Instruments ZetasizerNano ZS (UK). Fluorescence measurements were performed in a Varian Cary Eclipse spectrofluorometer (UK) and in a FLS920 series Edinburgh instruments spectrofluorometer (UK). For the surface plasmon resonance (SPR) experiments, L1 sensor chips and a Biacore X100 (Cytiva, UK) were used.

### Lipid vesicle preparation

Small unilamellar vesicles (SUVs) and large unilamellar vesicles (LUVs) composed of POPC : POPS (4 : 1) were used as membrane model systems. Lipids were solubilized in chloroform in a round-bottom flask and a gentle nitrogen flow was used to evaporate the organic solvent. The lipidic film was kept under vacuum overnight. Subsequently, it was rehydrated with 10 mM HEPES, 150 mM NaCl, pH 7.4 buffer followed by ten freeze/thaw cycles, forming a suspension of multilamellar vesicles. SUVs and LUVs were obtained by extrusion of the multilamellar vesicles through polycarbonate filters with pore size of 50 nm and 100 nm, respectively.

### Dynamic light scattering

Dynamic light scattering (DLS) was used to monitor lipid vesicle aggregation induced by the PIFP–F103A and PIFP–Q120A. The experiments were performed by successive additions of each peptide (covering final concentrations from 0.94 to 30 μM) to 0.77 mM POPC : POPS (4 : 1) LUVs, in 10 mM HEPES, 150 mM NaCl, pH 7.4 buffer. An incubation of 5 minutes at 37 °C was performed after each addition. Additionally, samples containing only the PIFP–F103A or PIFP–Q120A at the higher concentration tested were analysed as a control to ensure no PIFPs aggregation was detected. Three independent experiments were performed, each corresponding to an average autocorrelation curve obtained from at least ten replicate sample scans. The CONTIN method^15^ was applied to determine the diffusion coefficient (*D*) values of the vesicles of each sample. *D* values were then used to calculate the particles’ *Z*-average hydrodynamic diameter through the Stokes–Einstein–Sutherland equation.^16^

### Surface plasmon resonance

PIFP–F103A and PIFP–Q120A partition into lipid membranes was evaluated using SPR. L1 sensor chips were used throughout the experiments and were rinsed with three injections of 20 mM CHAPS before each assay. The lipid membrane surface was prepared with 1 mM POPC : POPS (4 : 1) SUVs in 10 mM HEPES, 150 mM NaCl, pH 7.4 buffer injected at a 2 μL min^−1^ flow rate for 2400 s. Then, a 36 s injection of 10 mM NaOH at 50 μL min^−1^ was performed to remove loosely bound vesicles. Peptide samples were prepared in 10 mM HEPES, 150 mM NaCl, pH 7.4 buffer, in concentrations ranging from 2.5 to 40 μM, and injected over the lipid surface at a 5 μL min^−1^ flow rate during 500 s (association time). For each sample a 1000 s dissociation time was allowed. After each run, the L1 sensor chip was regenerated with sequential injections of 20 mM CHAPS (5 μL min^−1^ for 60 s), 0.5% (v/v) SDS (5 μL min^−1^ for 60 s), 10 mM NaOH with 20% (v/v) methanol (50 μL min^−1^ for 36 s), and 10 mM NaOH (50 μL min^−1^ for 36 s). The assay was conducted at 25 °C and three independent experiments were performed. After correcting the response values for each peptide molecular weight, the partition coefficients (*K*_P_) were calculated using the following equation:1.1
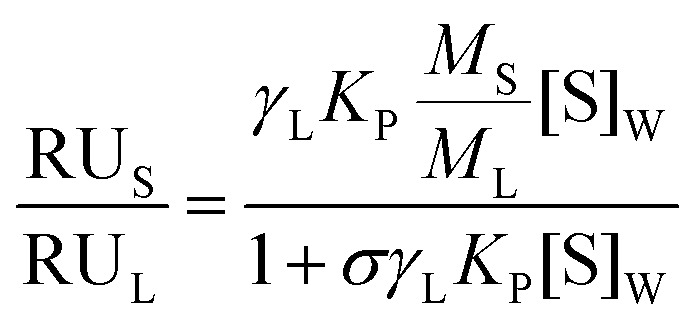
where RU_S_ is the total sample response, RU_L_ corresponds to the total lipid deposition response, *γ*_L_ is the lipid molar volume, *M*_S_ represents the peptide molecular weight, *M*_L_ is the lipid vesicles molecular weight, [S]_W_ is the sample concentration and *σ* corresponds to the lipid to solute ratio at the saturation point.

### Lipid mixing

Lipid mixing induced by the PIFP, PIFP–F103A, and PIFP–Q120A was evaluated by a fluorescence resonance energy transfer (FRET) approach. POPC : POPS (4 : 1) LUVs labelled with 1% N-NBD-PE (donor) and 1% N-Rh-PE (acceptor), and unlabelled LUVs were mixed in a 1 : 4 ratio in 10 mM HEPES, 150 mM NaCl, pH 7.4 buffer and used throughout the experiment. Successive additions of each peptide to a 0.77 mM LUVs sample were performed, covering a range of final concentrations from 2.5 to 20 μM. Fluorescence emission spectra were collected between 500 and 650 nm with the excitation wavelength (*λ*_exc_) at 470 nm, after a 15-minute incubation at 37 °C. The fusion efficiency (*R*) was determined as:1.2
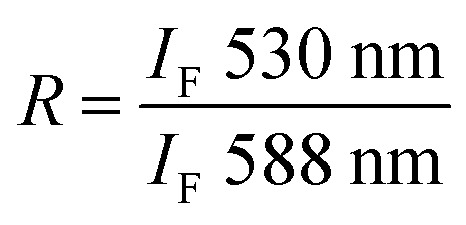
where *I*_F_ 530 nm is the fluorescence emission intensity recorded at 530 nm and *I*_F_ 588 nm is the fluorescence emission intensity recorded at 588 nm. Lipid mixing (%) was then determined using the following equation:1.3
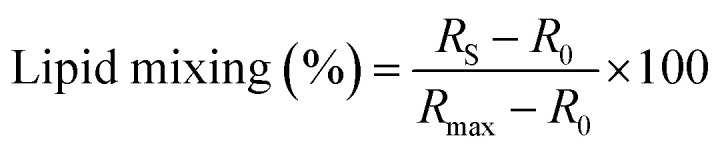
where *R*_S_ is the *R* of the sample, *R*_0_ is the *R* of the negative control—corresponding to the lipid vesicles in the absence of peptide—and *R*_max_ is the *R* for the positive control—corresponding to the lipid vesicles in the presence of 1% (v/v) Triton X-100. For each peptide, three independent experiments were performed.

### Vesicle content leakage

The content leakage from POPC : POPS (4 : 1) vesicles induced by the PIFP, PIFP–F103A, and PIFP–Q120A was determined by 5,6-carboxyfluorescein (5,6-CF) dequenching using LUVs loaded with 50 mM 5,6-CF as described elsewhere.^17^ Briefly, peptides, at final concentrations ranging from 0.94 to 20 μM, were incubated with 0.77 mM LUVs in 10 mM HEPES, 150 mM NaCl, pH 7.4 buffer for 15 minutes at 37 °C. Then, fluorescence emission intensity at 520 nm (*λ*_exc_ = 493 nm) was measured and the vesicle content leakage percentage was determined as followed:1.4
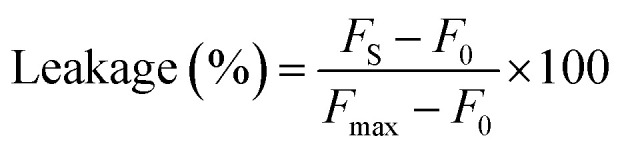
where *F*_S_ is the fluorescence emission intensity measured after the 15 minutes incubation of each peptide with the LUVs, *F*_0_ is the negative control, corresponding to the fluorescence emission intensity of the LUVs measured in the absence of peptide, and *F*_max_ is the positive control, corresponding to the fluorescence emission intensity measured after the addition of 1% (v/v) Triton X-100.

### Coarse-grain MD simulations

Solid-state NMR studies of PIFP revealed that the peptide adopts a fully α-helical conformation inside POPC:POPG membranes.^4^ Based on these data, a fully α-helical PIFP was created using the remodeling tool from Rosetta^18^ and converted to a Martini coarse-grained topology^19,20^ with the martinize 2.0 program.^21^ This program was also used to generate the topology and structure files based on the atomistic pdb file.

Triplicate coarse-grained (CG) simulations of the PIFP, PIFP–F103A and PIFP–Q120A were performed in a POPC : POPS membrane (at 80 : 20 molar ratio). To mimic conditions of high peptide concentrations, each system was set up with one peptide for every 70 lipids, resulting in an average of six peptides and 420 lipids per simulation box. The N-terminus of the peptides was considered protonated and the C-terminus considered neutral, to reproduce the state of peptides in the context of the F protein and the experimental setup. Peptides were inserted in a transmembrane orientation, with the α-helical axes parallel to one another and separated by at least 3 nm. The system was built using the INSANE script,^22^ in a 10 × 15 × 14 nm box. The total charge of the systems was first neutralized with either Na^+^ or Cl^−^ ions, and then an additional 140 mM NaCl was added. All simulations were performed with the GROMACS 2020.3 package,^23^ using the Martini 3 force field^20^ and lipid topologies released with it.

NMR data on the position of the PIFP in a POPC:POPG membrane shows that it should stay vertically inside the membrane.^24^ Preliminary tests showed that, contrary to the experimental data, when using the Martini 3 model the PIFPs quickly left the transmembrane orientation to become surface adsorbed. This behaviour has been observed with other transmembrane peptides, and ascribed to an overly hydrophilic peptide character in Martini 3.^25,26^ To prevent this from happening we scaled down the backbone–water interactions of the peptide as suggested by others:^25,26^ in all, except the first and last three amino acid residues, the backbone–water interaction epsilon was lowered by 1 kJ mol^−1^. For simplicity, this was implemented atop the standard Martini 3 peptide topologies using virtual backbone beads overlaid on the affected backbone particles.

The systems were energy-minimized using the steepest-descent method for 5000 steps. In this step the peptide's backbone atoms were position-restrained using a force constant of 500 kJ mol^−1^ nm^−2^. Before performing production runs, an equilibration step was carried without any restraints for 20 ns, using the Berendsen barostat to regulate pressure. The simulations were run in the isothermal–isobaric (NPT) ensemble, coupling the system to a temperature and pressure bath using the v-rescale and Parrinello–Rahman^27^ schemes, respectively. The target temperature was set to 310 K and semi-isotropic pressure coupling was set to a target pressure of 1 atm using a compressibility of 3 × 10^−4^ bar^−1^. Reaction-field electrostatics were employed, and Lennard-Jones and coulombic interactions were both cut-off at 1.1 nm.^28^ Each system was run for 30 μs.

### Atomistic MD simulations

Atomistic MD simulations of the PIPF, PIFP–F103A and PIFP–Q120A were performed using the last frame of the CG simulations as the starting structure. The systems were converted from coarse-grain to atomistic detail using the backward.py script.^29^ All atomistic simulations were performed with the GROMACS 2020.3^23^ package and using the Amber14sb forcefield,^30^ alongside the TIP3P water model.^31^ The system was energy-minimized for 5000 steps using the steepest-descent algorithm. Following minimization, equilibration was performed. The system was coupled to a Nosé–Hoover thermostat^32^ to maintain a temperature of 310 K with a coupling constant of 1 ps, and semi-isotropic pressure coupling was applied using a Parrinello–Rahman barostat.^27^ During the equilibration, atomic position restraints on the protein heavy atoms were gradually relaxed in six steps from 4000 to 2000 kJ mol^−1^ nm^−2^ and from 500 to 200 kJ mol^−1^ nm^−2^ on the protein backbone and side-chains, respectively. The lipid's phosphate atoms were also gradually relaxed from 1000 to 40 kJ mol^−1^ nm^−2^. After the sixth step, production simulations were performed.

Simulations were run in the isothermal–isobaric (NPT) ensemble, coupling the system to a temperature and pressure bath using the Nosé–Hoover^32^ and Parrinello–Rahman^27,33^ schemes, respectively. The target temperature was set to 310 K and semi-isotropic pressure coupling also set to 1 atm using a compressibility of 4.5 × 10^−5^ bar^−1^. Long-range electrostatic interactions were treated with the PME^33^ scheme, using a grid spacing of 0.12 nm, with cubic interpolation. The neighbour list was updated every twenty steps with a Verlet cutoff with a 1.2 nm radius. Rigid bonds involving hydrogen atoms were constrained using the LINCS algorithm.^34^ Three replicates were simulated for each system with all simulations being run for 1.5 μs.

### MD simulations analysis tools

To analyse the atomistic simulation data obtained we started by calculating the interpeptide interactions between the FPs in the membrane using RIP-MD,^35^ 250 frames from each replicate of all systems were analysed. Hydrogen bonds and π-stackings with a prevalence higher than 30% and 5% of the simulation, respectively, were selected. The resulting residue interaction networks (RINs) were visualized using Cytoscape.^36^

To assess whether the substituted PIFP peptides allow for water to flow through the membrane, we analysed the available space within the membrane and calculated the number of water molecules that passed through the membrane on the final 1 μs of the atomistic simulations. For this we applied the HOLE method,^37,38^ and the fluxer.py script,^39^ respectively.

To investigate lipid tail protrusion induced by the peptides, a Python script was written to count lipids within 4 Å of the peptides, where at least one carbon atom in the lipid tail extended more than 0.1 nm above the phosphate headgroup. Finally, simulation snapshots were visualized and rendered using VMD.^40^

## Results and discussion

### F103A and Q120A substitutions do not affect the PIFP ability to induce lipid vesicle aggregation

This study builds on previous research that highlighted the importance of the Q120 residue in maintaining peptide–peptide interactions between the PIFPs when inside a membrane,^14^ and the ability of the N-terminal residue, F103, to induce lipid tail protrusion—an intermediate step of lipid mixing events.^11,41^ Despite this, a comprehensive understanding of the role of these amino acid residues in peptide–peptide and peptide–membrane interactions remains elusive. In this work, three PIFPs were studied: a PIFP comprising the sequence present in the fusion protein of the PIV5 virus, and two others with the F103A and Q120A substitutions, PIFP–F103A and PIFP–Q120A, respectively. Alanine, commonly used in assessing residue side chain roles, was chosen as a substitute because it induces minimal structural disruption of the peptide or protein—by removing the side chain beyond the β-carbon without adding stringent steric or electrostatic effects. Furthermore, alanine substitutions minimize disruptions on the overall peptide structure, unlike amino acid residues such as proline or glycine.^42^ The aim is to gain insight into the influence of the residues F103 and Q120 on the effect of PIFP on lipid membranes by comparing the results obtained with the PIFP–F103A and PIFP–Q120A with those that have been obtained for the PIFP.

**Fig. 2:**
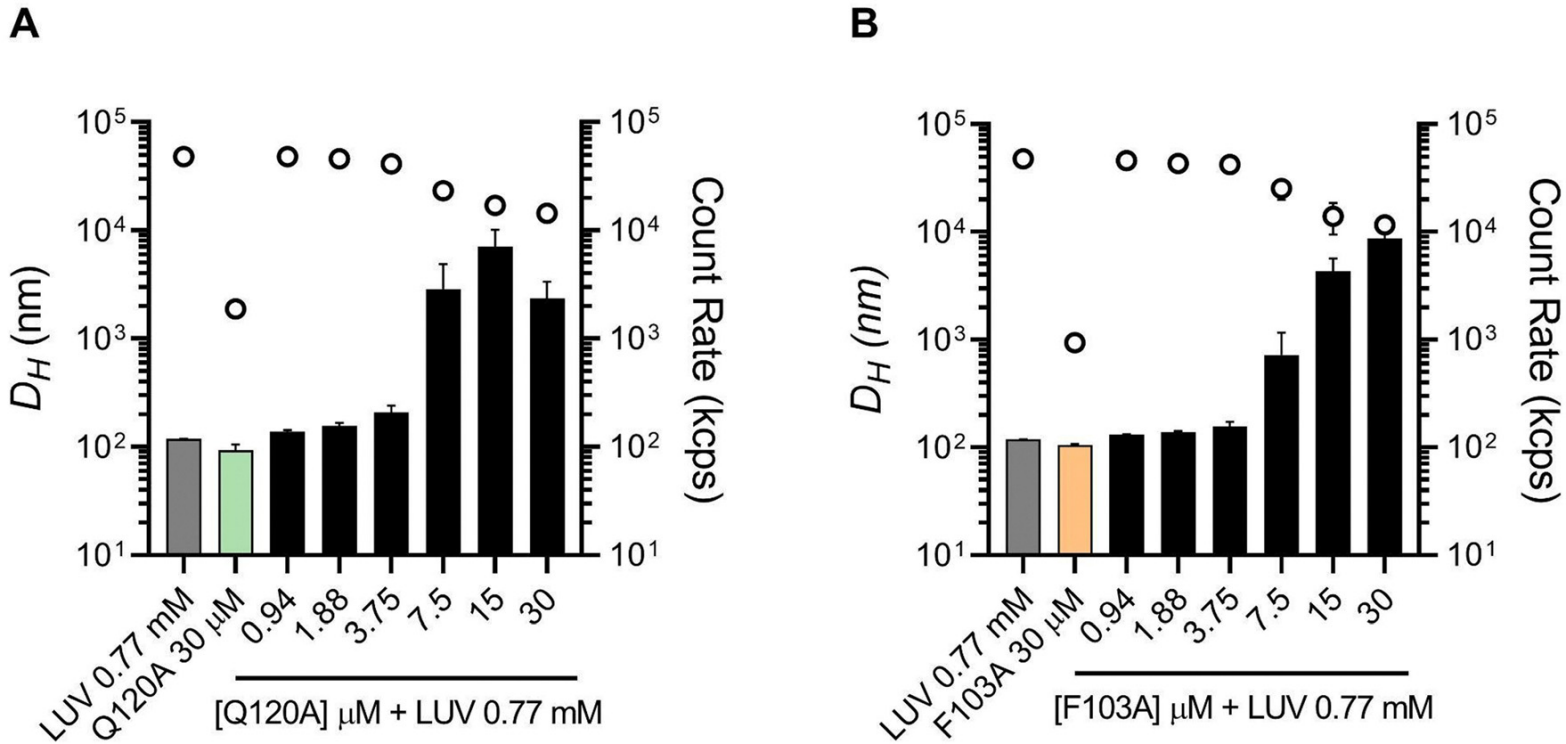
PIFPs and PIFPs-induced vesicle aggregation profiles. Monitorization with DLS of *Z*-average *D*_H_ (columns) and count rate (circles) of particles formed during (A) PIFP–Q120A and (B) PIFP–F103A titration in a LUVs solution. Aggregation profiles of LUVs or PIFPs alone were used as controls.

The oligomerization of viral FPs on the cell membrane surface is a shared feature among diverse viruses, including PIV. For the PIFP, we previously reported^11^ a concentration-dependent accumulation of the peptide on the surface of lipid vesicles and a consequent triggering of their aggregation. To investigate the influence of the Q120 and F103 residues on the ability of PIFP to induce lipid vesicle aggregation DLS was employed. The measurement of the hydrodynamic diameter (*D*_H_) as well as the particle count rate upon consecutive additions of the peptides to lipid vesicles allowed us to obtain insights into the aggregation process. For the PIFP–Q120A, an increase in peptide concentration from 3.75 to 7.5 μM results in a significant 24-fold increase in the lipid vesicles *D*_H_ and a 2-fold decrease in the particle count (Fig. 2A). Thus, the PIFP–Q120A presents a critical concentration of peptide for vesicle aggregation between 3.75 and 7.5 μM, similar to what was previously described for PIFP.^11^ With PIFP–F103A, a similar pattern is observed: as the concentration of PIFP–F103A increases from 3.75 to 7.5 μM, there is a 5-fold increase in the lipid vesicles’ *D*_H_ along a 1.7-fold decrease in particle count (Fig. 2B). These results show that, similarly to the PIFP, upon reaching a critical concentration of peptide for vesicle aggregation, between 3.75 and 7.5 μM, both PIFP–Q120A and PIFP–F103A trigger the aggregation of lipid vesicles, suggesting that the residues F103 and Q120 do not play a significant role in PIFP's ability to induce lipid vesicles aggregation.

### F103A and Q120A substitution in the PIFP alter its membrane affinity

**Fig. 3:**
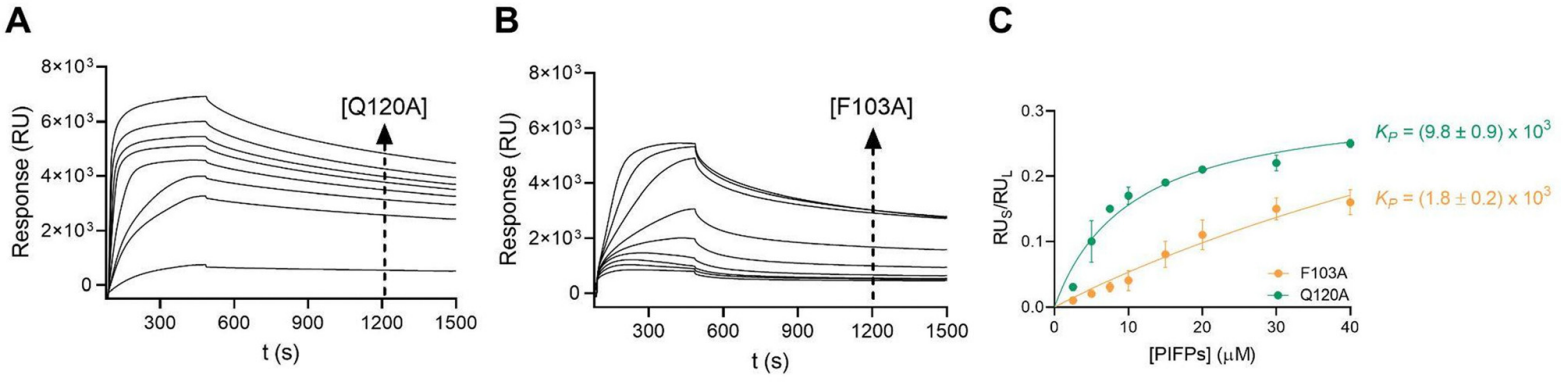
Characterization of PIFPs–lipid membrane interactions. Surface plasmon resonance (SPR) sensorgrams at increasing concentrations (2.5, 5, 7.5, 10, 15, 20, 30, and 40 μM) of (A) PIFP–Q120A and (B) PIFP–F103A. (C) *K*_P_ values were determined by representing the individual values of RU_S_ and RU_L_ recorded for each PIFP sensorgram at 500 s as the function of the corresponding concentration.

Given the ability of PIFP–Q120A and PIFP–F103A to induce lipid vesicle aggregation, it is important to characterize their lipid binding properties. To assess peptide–lipid binding, SPR was used, since this technique allows real-time detection of bound peptides on a surface coated with lipid vesicles. Each peptide's partition coefficient was estimated by plotting peptide-to-lipid response ratios as a function of the injected peptide concentration, as described previously.^43^ Although both peptides displayed an increase in lipid binding responses in a concentration-dependent manner, different partition coefficient (*K*_P_) values were obtained (Fig. 3). PIFP–Q120A exhibited a *K*_P_ of (9.8 ± 0.9) × 10^3^, which is higher than the *K*_P_ value reported for the PIFP (*K*_P_ = (3.2 ± 0.4) × 10^3^).^11^ In contrast, PIFP–F103A displayed a *K*_P_ of (1.8 ± 0.2) × 10^3^, which is lower than the *K*_P_ of either PIFP or PIFP–Q120A. These results show that the Q120A substitution does not compromise the peptide's ability to bind to the membrane, and even increases the peptide's partition coefficient, possibly due to the higher hydrophobic nature of the peptide after the Q120A substitution.^44^ Conversely, the F103A substitution reduces the peptide's affinity for the lipid membrane, demonstrating the importance of the N-terminal Phe for the insertion of the PIFP into the membrane bilayer.

The local concentration of each peptide in vesicle membranes was calculated, using the partition coefficient and as a function of the peptide concentration, as described by Castanho *et al.*^45^ The calculated values are listed in Table 1. Following the trend in *K*_P_ values, for a given concentration of lipid and peptide the local concentration of PIFP–F103A in vesicle membranes will be lower than that of PIFP, which in turn will be lower than that of PIFP–Q120A. This highlights an important role of the F103 residue in mediating the interaction of PIFP with membranes since its substitution significantly reduces (by approximately 20%) the peptide's association with lipid vesicles.

**Table 1:** The local concentration of each peptide in the lipid vesicle membranes ([P]_M_) was calculated considering the total peptide concentration ([P]_T_), the partition coefficient, and a lipid concentration of 1 mM, as described in ref. 42

[P]_T_ (μM)	[P]_M_ (mM)		
	PIFP	PIFP–F103A	PIFP–Q120A
0.94	0.9	0.7	1.1
1.88	1.7	1.4	2.2
2.50	2.3	1.9	2.9
3.75	3.5	2.8	4.3
5.00	4.7	3.8	5.8
7.50	7.0	5.7	8.7
10.00	9.3	7.6	11.6
15.00	14.0	11.4	17.3

### F103 is critical for the PIFP's ability to induce lipid mixing and membrane leakage

To explore the effect of the selected substitutions on the PIFP fusogenic properties, we examined PIFP–Q120A and PIFP–F103A's ability to induce both lipid mixing and vesicle leakage. Lipid mixing was monitored by peptide-induced FRET efficiency variations in a mixture composed of NDB-RhB-labelled and unlabelled vesicles while vesicle leakage was evaluated by monitoring the peptide-induced leakage of 5,6-carboxyfluorescein (5,6-CF)-loaded vesicles.

**Fig. 4:**
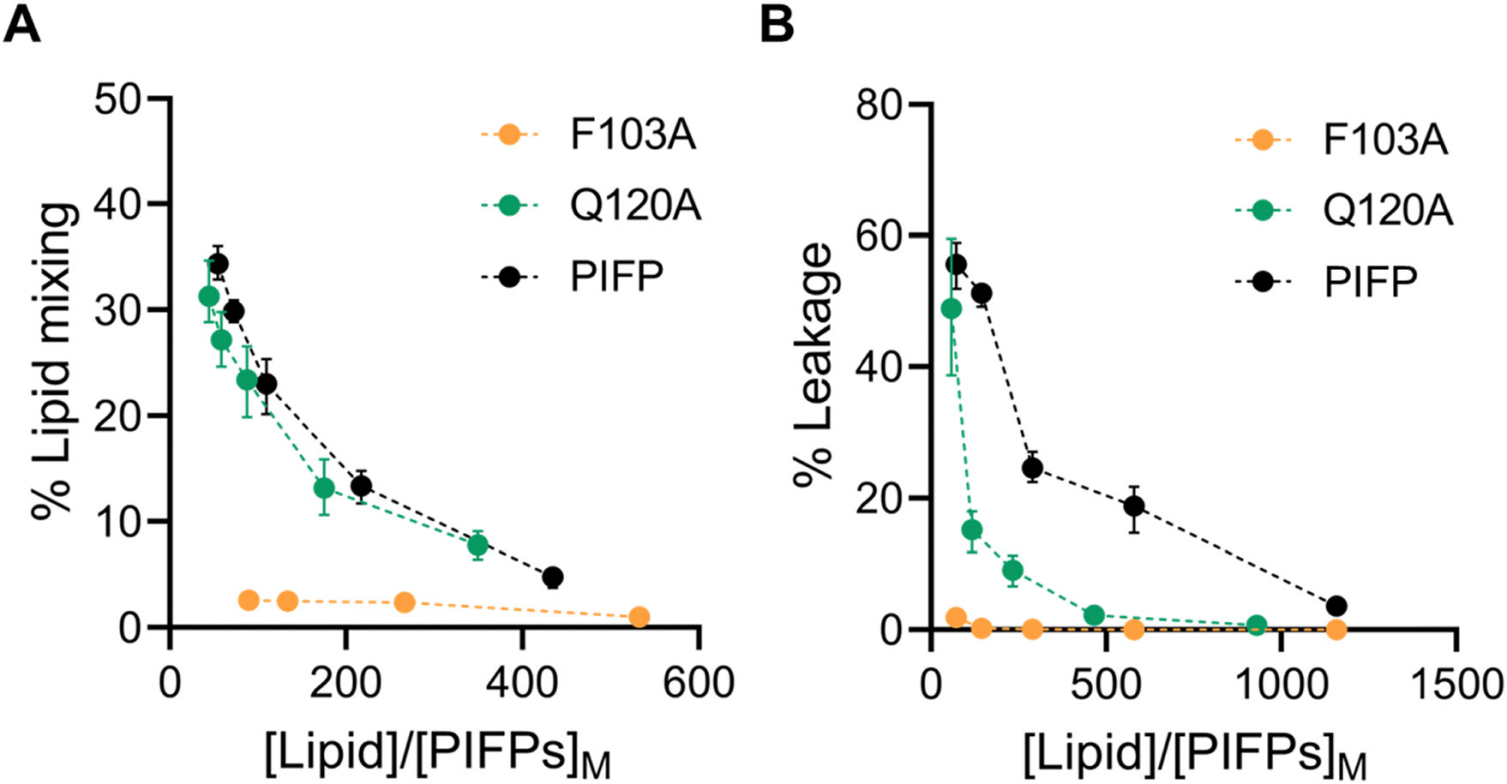
Characterization of PIFPs fusogenic properties. Percentage of PIFPs-induced lipid mixing (A) and percentage of PIFPs-induced membrane leakage (B) at increasing ratio of lipid to peptide concentrations in the membrane. (Black dots, PIFP; green dots, PIFP–Q120A; orange dots, PIFP–F103A.) Error bars were calculated with bootstrap resampling and correspond to the 95% confidence intervals.

Interestingly, the Q120A and F103A substitutions impacted lipid mixing and vesicle content leakage to different degrees. F103A essentially lost the ability to induce lipid mixing (Fig. 4A) or membrane leakage (Fig. 4B). In Q120A, however, the substitution had no significant effect on the peptide-induced lipid mixing (Fig. 4A) and yielded a membrane leakage lower than that of native PIFP but still measurable (Fig. 4B).

The above findings show that substituting the N-terminal phenylalanine has a significant impact on the membrane perturbation properties of the PIFP, while the substitution of the glutamine residue only affects the ability of the peptide to induce membrane leakage. From these results, it seems clear that both lipid mixing and leakage ability depend on separate factors: the peptide concentration within the membrane and the influence of peptide–peptide interactions on membrane disruption.

On the one hand, PIFP–F103A exhibits a significantly reduced partition coefficient relative to both PIFP and PIFP–Q120A. This reduced partitioning likely prevents effective membrane insertion, impairing the ability of PIFP–F103A to induce the membrane perturbations required for lipid mixing or vesicle content leakage. Furthermore, as an aromatic residue, F103 may contribute to π-stacking and hydrophobic interactions that help anchor the peptide in the membrane and promote its destabilization. These interactions may position the peptide in a conformation that facilitates the assembly of oligomeric porelike structures necessary for vesicle content leakage. In the absence of F103, these anchoring and destabilizing interactions are disrupted, preventing both lipid mixing and leakage, even when Q120 is still present.

On the other hand, the Q120A substitution maintains effective membrane insertion and promotes lipid mixing, indicating that Q120 is not essential for initial membrane interaction. However, the observed reduction in vesicle content leakage suggests that Q120 plays a role in stabilizing the peptide–peptide interactions required for the formation of the porelike structure and vesicle content leakage. The reduction in vesicle leakage can also be attributed to the substitution of the polar glutamine with a more hydrophobic residue that reduces the propensity of water molecules to pass through the membrane.

These results indicate that F103 and Q120 contribute differently to PIFP's membrane activity, with F103 playing a broader role in membrane interaction and Q120 being more specifically involved in interpeptide interactions and leakage. Because validating this hypothesis *in vitro* would impose significant challenges, we followed an *in silico* approach to further explore this matter.

### MD simulations reveal that F103A and Q120A assemble into oligomeric structures stabilized by distinct interaction networks

To compare and validate our experimental results, we conducted MD simulations of multiple PIFPs in POPC:POPS membranes. Simulations were started with the peptides already in a transmembrane configuration. This was supported by prior solid-state NMR data, which show that the PIV5 fusion peptide forms a stable transmembrane helix bundle within the membrane, even with a glutamine residue near the membrane core.^24^ Additionally, attempting to simulate spontaneous insertion from solution would be computationally prohibitive due to the large conformational space that needs to be sampled.

Although the PIFP–F103A and PIFP–Q120A substitutions could, in principle, impact the insertion of the peptides, we designed the simulations assuming that these peptides would also be in transmembrane orientation. Despite the decrease in partition observed experimentally, here we focused on comparing the effects of the different peptide variants once inserted in the membrane, aiming to dissect the impact of the F103A and Q120A substitutions on the fusion peptide's ability to interact with and perturb the membrane. Nevertheless, even with the F103A substitution the PIFP–F103A continues to have an affinity to the membrane and maintain its hydrophobic character. Therefore, it is reasonable to assume that it can adopt a “membrane-spanning configuration”. The Q120A substitution should further facilitate transmembrane insertion, as a polar residue in the middle of the helix, and close to the membrane core, is being replaced by a hydrophobic one.

**Fig. 5:**
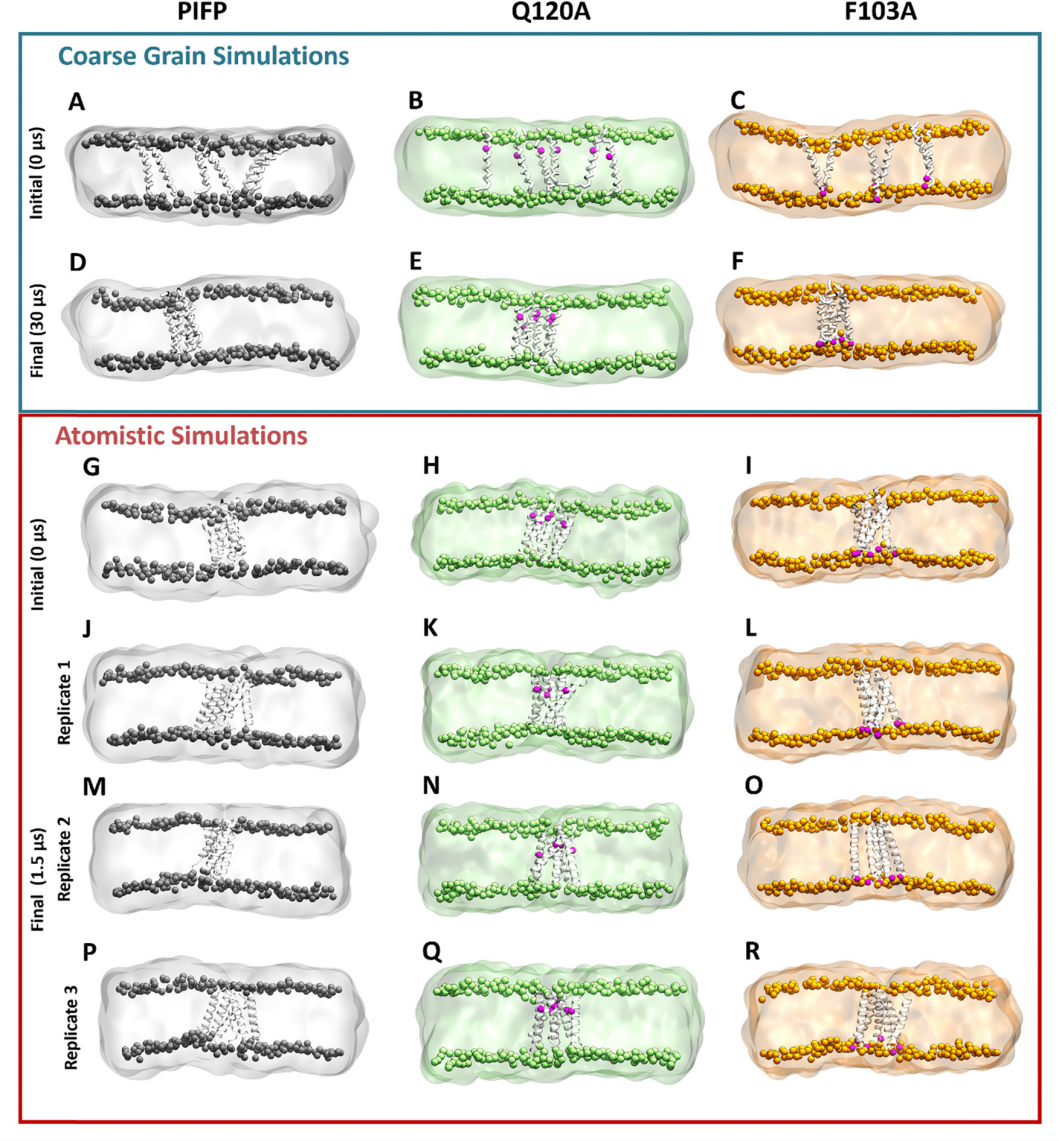
Snapshots of the initial and final frames of the CG and atomistic MD simulations. Initially, six PIFPs were inserted into a POPC : POPS (4 : 1) membrane (A)–(C). The three systems were then simulated in CG detail for 30 μs, with peptides aggregating inside of the membrane (D)–(F). The last frame of the CG simulation was converted to atomistic detail recurring to the backward tool^26^ (G)–(I). Three independent replicates of each system were then simulated for 1.5 μs (PIFP: J, M and P; Q120A: K, N and Q; F103A: L, O and R). Lipids belonging to the PIFP, Q120A and F103A systems are shown in gray, green and orange, respectively. Substitution sites Q120A and F103A are shown in magenta.

During the CG simulations, all peptides aggregate in the membrane (as seen in Fig. 5D–F) as transmembrane bundles. These structures were used as the initial conformations for atomistic MD simulations (Fig. 5G–I).

**Fig. 6:**
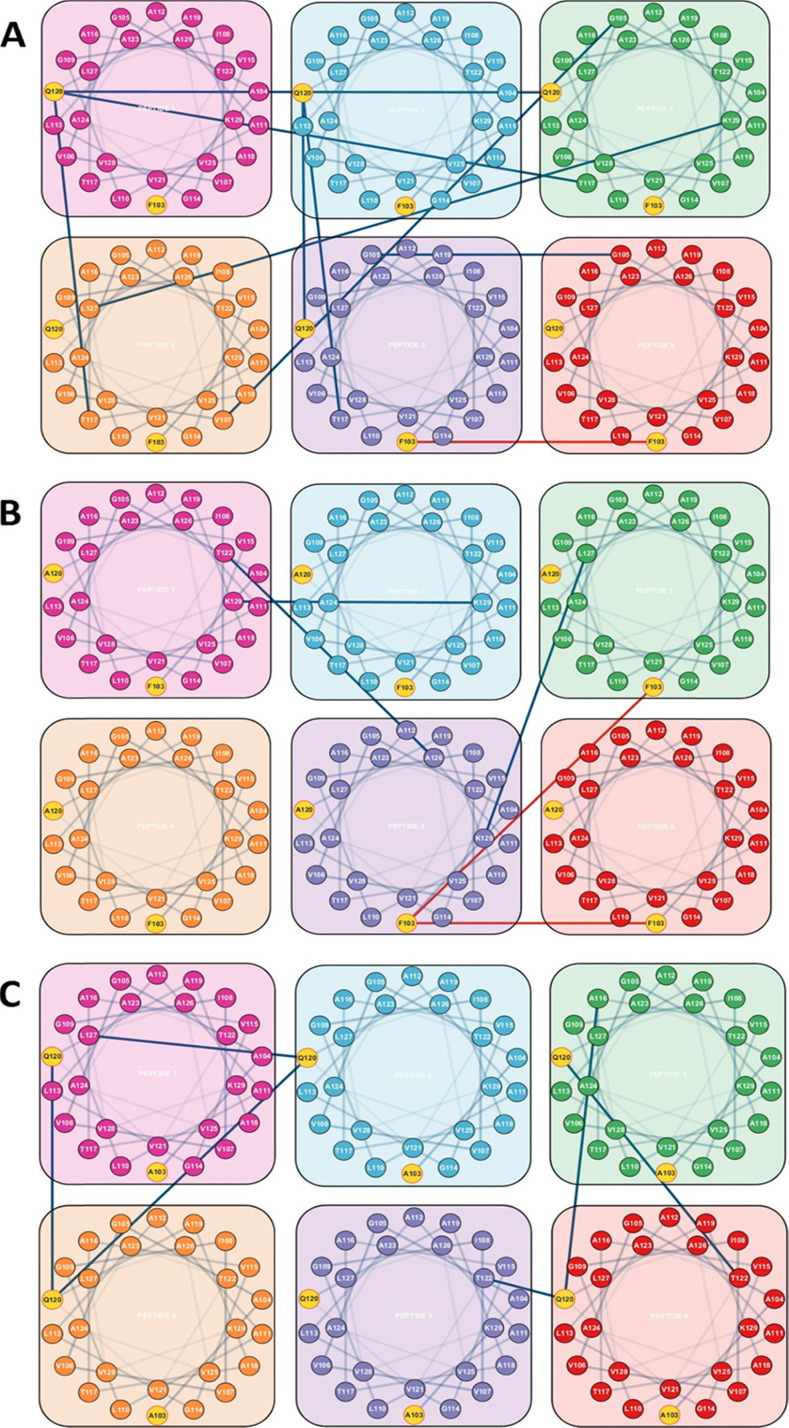
Residue interaction networks (RINs) for the peptide–peptide interactions of the (A) PIFP, (B) PIFP–Q120A and (C) PIFP–F103A systems. The RINs were determined using RIP-MD. Hydrogen bonds with prevalence over 30% are shown in blue and π-stacking interactions with prevalence over 5% are shown in red.

Upon visual inspection of the systems after 1.5 μs of simulation at atomistic resolution, it is evident that all peptides remain embedded within the membrane, and that the PIFP aggregates persist, as seen in the lower section of Fig. 5, supporting the assumption that the peptides are stably inserted in the membrane. However, this initial analysis, while informative, does not provide a comprehensive understanding of the true influence of the substitutions on the aggregated structures. To achieve this, we started by analysing the impact of the single substitutions on the formation of interpeptide interactions. The RIP-MD tool was used,^35^ and 250 frames of each replicate were analysed. All hydrogen bonds and π-stacking interactions were calculated, and the residue interaction networks (RINs) for the peptide–peptide interactions were plotted using Cytoscape^36^ (Fig. 6). In the PIFP system, we observed the presence of eight persistent hydrogen bonds, found in more than 30% of the frames analysed. The majority of these hydrogen bonds were mediated by Q120 and T117 residues. In addition to the side chain-mediated hydrogen bonds involving glutamine and threonine, backbone interactions were also observed. Specifically, G105 formed hydrogen bonds with itself or with V107. Besides the hydrogen bonds, we also observed a less persistent pi-stacking interaction, present in approximately 10% of the simulation frames analysed, involving the N-terminal phenylalanines (F103) of peptides 5 and 6 (Fig. 6A).

When replacing Q120 by alanine, a noticeable decrease in the overall number of interactions was observed, from eight to three. There was a complete abolishment of interactions mediated by the substituted A120 residue, as depicted in Fig. 6B, while new hydrogen bonds emerged involving residues K129, T122, A126, and L127, with a persistence higher than 30% of the analysed frames. Specifically, we observed the formation of the following inter-peptide hydrogen bonds: T122–A126, K129–K129, and L127–K129. Regarding the π-stacking interactions, a new interaction between two F103 residues appeared, although its persistence remained low, at approximately 5%.

Finally, in the system where the F103 amino acid residue was substituted with an alanine residue, the π-stacking interactions previously mediated by this residue ceased to exist. Interestingly, the number of persistent inter-peptide hydrogen bond interactions increased compared to the PIFP–Q120A system, going from three to six. However, this is still lower than the eight hydrogen bond interactions observed in the PIPF. In the PIFP–F103A system the Q120 amino acid residue played a crucial role, by mediating all observed peptide–peptide interactions. These include the pairs Q120A–Q120A, Q120A–T122, Q120A–L127, and Q120A–A116.

In conclusion, the introduction of the PIFP–Q120A and PIFP–F103A substitutions had distinct impacts on the peptides’ ability to oligomerize inside the membrane. The Q120A substitution led to a significant reduction in overall interactions, including the complete abolishment of interactions mediated by the new A120 residue. While new hydrogen bonds formed, they remained fewer than in the PIFP system, indicating the crucial role of Q120 in maintaining peptide–peptide interactions and oligomerization inside the membrane. On the other hand, the F103A substitution eliminated π-stacking interactions but increased hydrogen bond interactions compared to the PIFP–Q120A system. However, even with Q120 still playing a pivotal role in mediating interactions, these were still fewer than in the native PIFP system. These results underline the importance of specific amino acid residues, particularly Q120, in stabilizing and modulating the interactions of these peptides within the membrane, allowing for their oligomerization.

### Q120A and F103A substitutions lead to a decrease in water flux through the membrane

The oligomerization behaviour of the PIFPs in the membrane, seen here, was also shown by us in a previous study.^11^ Our earlier observations strongly suggested that peptide oligomerization inside the membrane played a pivotal role in promoting the formation of water-permeable porelike structures. To determine whether these substituted peptides would also allow for the flow of water through them, we looked at the available space inside the membrane and calculated how many water molecules could pass through the pore formed by the peptides per time unit (nanoseconds – ns). To do this, we examined the last 1 μs of the atomistic simulations using the HOLE method,^37,38^ and the fluxer.py script.

**Fig. 7:**
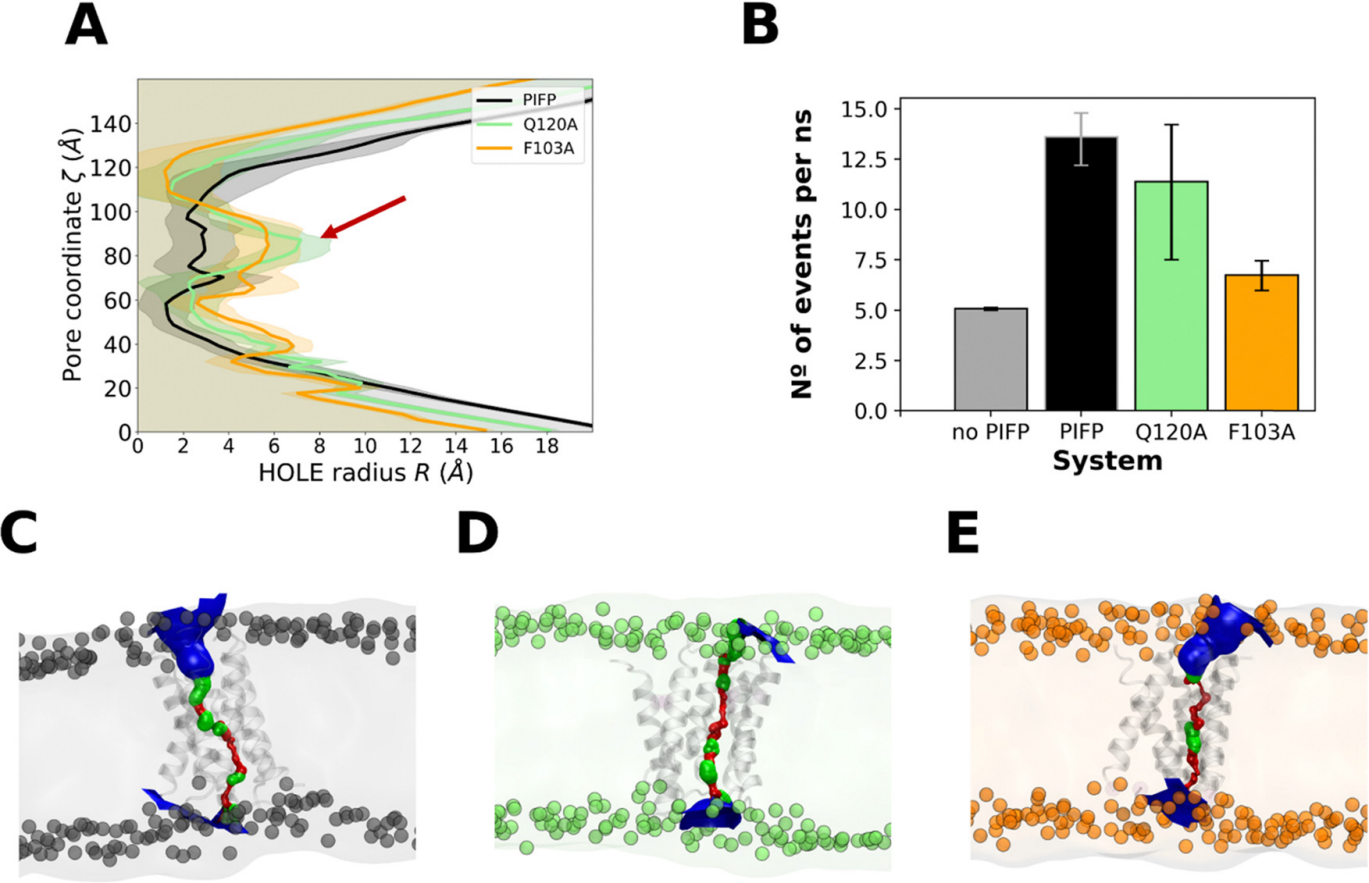
PIFP pore characterization. Graph (A) shows the radius of the pores for system PIFP (in black), PIFP–Q120A (in green) and PIFP–F103A (in orange). The pore radius was determined using the spherical probe of the HOLE algorithm.^34,35^ Below the curves, the average space available for water passage is highlighted. Panel (B) displays a snapshot from the PIFP, PIFP–Q120A and PIFP–F103A simulations, clearly illustrating the space available in the membrane for the passage of water. Error bars were calculated with bootstrap resampling and correspond to the 95% confidence intervals. Panels (C)–(E) are representative snapshots generated with the HOLE2 analysis tool from MD analysis that show the accessibility of water to the membrane for the PIFP, PIFP–Q120A and PIFP–F103A systems, respectively.

Knowing that a radius of at least 1.15 Å is necessary to accommodate a water molecule, our results show that, on average, all the systems studied form channels sufficiently large to allow water molecule passage along their whole length (Fig. 7A and C–E). This spacing is even wider in the region close to the substitution site Q120A, as shown in Fig. 7A by the red arrow. This observation is also in line with what is shown in Fig. 6B, that when Q120 is replaced by alanine a notable decrease in the overall number of interactions is observed and no new interactions are formed by the substituted A120 residue.

Looking at the average number of waters crossing the membrane per time unit, we see that the flux (measured in molecules ns^−1^) through the PIFP pore is the highest, followed by the PIFP–Q120A system, and finally the PIFP–F102A system (Flux_PIFP_pore = 12.65 ± 2.94 > Flux_Q120_pore = 8.83 ± 4.58 > Flux_F103A_pore = 6.75 ± 1.53).

Overall, the PIFP system exhibits the highest water flux through the pore, followed by the PIFP–Q120A and PIFP–F103A systems. The same trend was seen in the biophysical assays, where the introduction of the glutamine substitution (Q120A) results in a decrease of 5,6-CF leakage, which is even more impacted when substituting the N-terminal phenylalanine (Fig. 4B). Nevertheless, in the leakage assays the substitution of the N-terminal phenylalanine—F103—led almost to the abolition of leakage, whereas in computational results some flux is still observed through the porelike structure. This discrepancy between *in silico* and *in vitro* experiments may be explained by a fundamental difference in the setup of the simulations compared to the biophysical assays. In the computational systems, the PIFPs were initially inserted in a transmembrane manner, as described by PIFP NMR structure data.^24^ Yet, in the experimental setup, the peptides are initially placed in solution and are incorporated into the membrane based on their propensity, *i.e.* the *K*_P_. In fact, the experimental data show that the PIFP–F103A has a *K*_P_ for the membrane 3-fold and 5.4-fold lower than that of the PIFP or PIFP–Q120A systems, respectively (Fig. 3C). This discrepancy can be attributed to the substitution of an aromatic residue, commonly found in viral FPs, which has been demonstrated to play a crucial role in aiding the peptide's insertion into the membrane.^46,47^ Consequently, this observation could suggest that our assumption that F103A is transmembrane may not be entirely accurate, implying that F103 could alternatively function as a hydrophobic anchor.

Additionally, the differences observed between *in silico* and *in vitro* results may be explained by the size disparity between water and 5,6-CF. While water has a molecular diameter of ∼3 Å, 5,6-CF is considerably larger, with an estimated diameter of 10–12 Å. Given that the average radius at the narrowest point of the PIFP-induced pores is ∼1.5 Å (Fig. 7A), water molecules can permeate through these pores, whereas the larger 5,6-CF is likely excluded. Although the minimum pore radius is similar across all peptide variants, the presence of a polar residue in the acyl chain core region may facilitate water passage in the case of PIFP, while hindering it in PIFP–Q120A.

All together the trends observed in both experimental (Fig. 4B) and *in silico* assays (Fig. 7B) converge, indicating that both substitutions impact the passage of 5,6-CF or water through the membrane. The F103A substitution stands out by significantly reducing the passage of content through the membrane.

### Substitution of the N-terminal phenylalanine residue significantly affects lipid tail protrusion

**Fig. 8:**
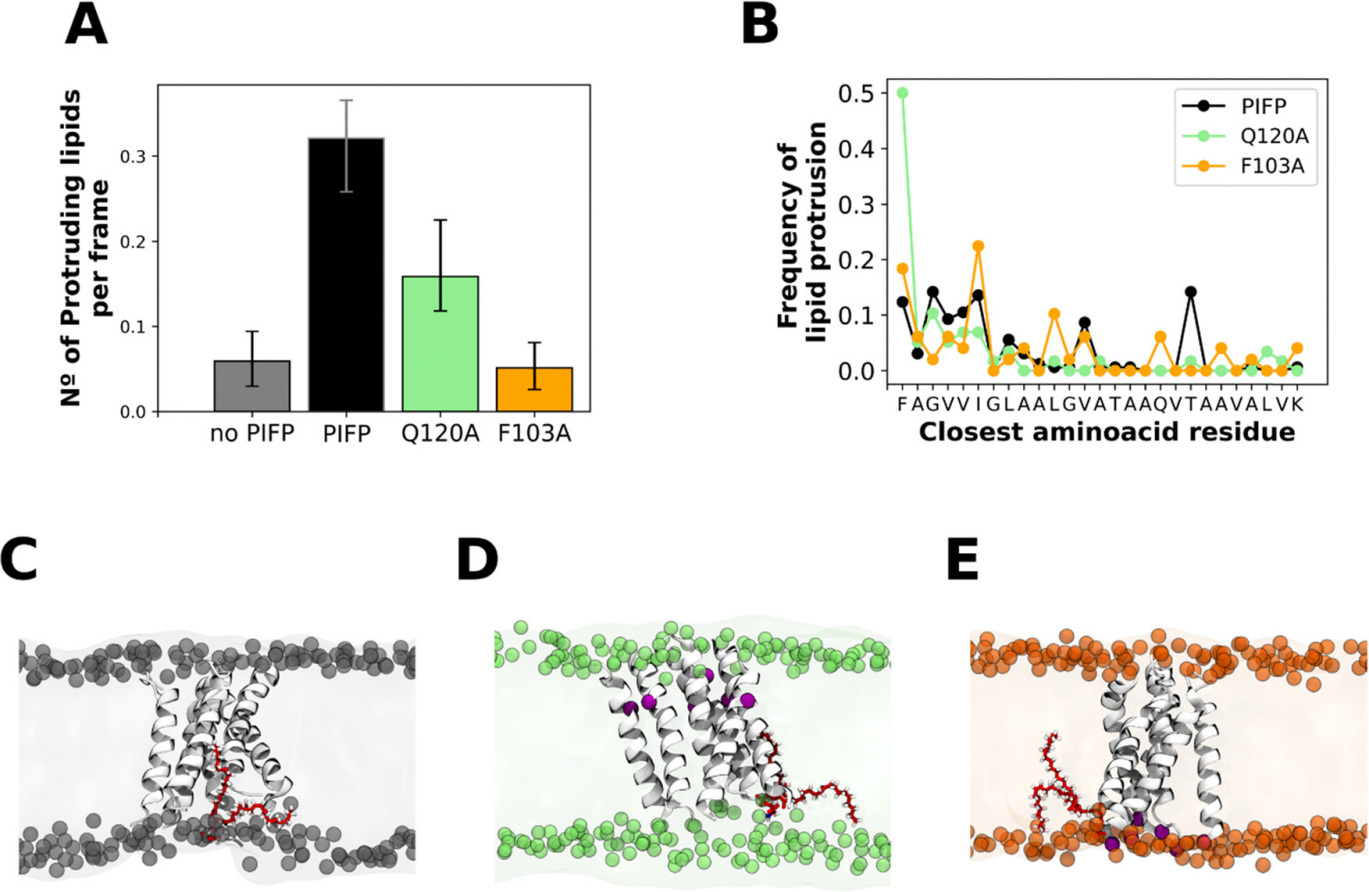
Lipid tail protrusion. (A) The plot shows the average number of protruding lipids at 4 Å from the peptides. Error bars represent the 95% confidence intervals and were calculated with bootstrap resampling. Plot (B) shows the amino acid residues that are closest to a protruding lipid. Panels (C)–(E) are snapshots of the PIFP, PIFP–Q120A and PIFP–F103A simulations, respectively, where a lipid is shown in the membrane context as a representation of a lipid tail protrusion event. The substitution sites are highlighted in red.

The passage of water through a membrane represents an atypical event given the membrane's hydrophobic nature. Our observation that upon the addition of these peptides, there is the passage of water through them, indicates that they must significantly perturb the lipid bilayer. To evaluate this, we analysed one of the computational hallmarks of membrane perturbation: lipid tail protrusion.^48–50^ This phenomenon was initially identified through MD data and is characterized by the outward extension of lipid acyl chains beyond their corresponding phosphate head groups (as depicted in Fig. 8C–E).

To measure the membrane disturbance induced by the PIFPs we determined the number of membrane lipid tail protrusion events occurring in the last 1 μs of the atomistic simulations. Results show that both substitutions lead to a decrease in the number of lipid acyl protrusion events (Fig. 8A), with the F103A substitution leading to the lowest number of lipid tail protrusion events (Fig. 8A). Upon analysing the amino acid residues near the lipid protrusion sites, we observed that the A103 substitution significantly reduces the frequency of lipid-tail protrusions close to the N-terminal residue when compared with both the PIFP and PIFP–Q120A systems (Fig. 8B). For the PIFP–Q120A system, a closer examination of the amino acid residues near the lipid protrusion sites revealed that the substitution of Q120 to an alanine leads to an abolishment of the lipid tail protrusion events induced by this residue when compared with PIFP (Fig. 8B).

These results are commonly correlated with lipid mixing due to the known effect of lipid tail protrusion in facilitating fusion between opposing membranes.^50–55^ In this study, both the biophysical and *in silico* assays point to a lower lipid mixing induced by PIFP–F103A, whereas in the case of the PIFP–Q120A the integration of the computational and experimental results is not so straightforward. Computational results show a lower induced lipid tail protrusion by PIFP–Q120A when compared with PIFP (Fig. 8A), whereas experimental results show no significant differences in lipid mixing (Fig. 4B). Nevertheless, when looking at the trends, the lipid mixing induced by the PIFP–Q120A is consistently lower than the PIFP and higher than the PIFP–F103A.

In summary, the trends seen for the passage of molecules through the membrane align with those observed for the membrane perturbation abilities of the PIFPs. Similarly, to the leakage results, both substitutions lead to a reduction of lipid tail protrusion events, with the F103A substitution having the most significant impact.

## Conclusion

In this study, we investigate the impact of specific substitutions on the ability of PIFP to interact with and disturb membranes. This was achieved by combining a range of experimental biophysical techniques with computational simulations to gain valuable insights into how specific substitutions affect PIFP properties.

We found that the Q120A substitution does not significantly impact the peptide's ability to induce vesicle fusion. This is supported by its similar lipid mixing and vesicle aggregation capabilities when compared to the PIFP. *In silico* simulation results also align with this trend. We hypothesize that the higher hydrophobicity of the alanine residue contributes to its enhanced membrane insertion capability. Simultaneously, this substitution significantly affects the formation of peptide–peptide interactions within the membrane and leads to a reduced water flux through the membrane.

In contrast to the PIFP–Q120A peptide, for the N-terminal phenylalanine substituted peptide (PIFP–F103A), we observe a significant reduction in the peptide's ability to induce vesicle fusion. Both lipid mixing and membrane leakage are nearly abolished in contrast to the PIFP or PIFP–Q120A systems. This drastic decrease is partially due to the lower partition coefficient of this peptide to the membrane, nearly two times lower than that of the PIFP and five times lower than that of PIFP–Q120A. This could be attributed to the fact that an aromatic amino acid residue is being substituted. These residues are commonly found in viral FP and have been shown to play a vital role in helping the peptide insertion into membranes.^46,47^ Another important feature of aromatic residues is their ability to interact favourably with membrane phospholipids and to partition at the lipid tail–lipid head interface, and this interaction can help destabilize the membrane and promote bilayer fusion.^47^ Furthermore, the fact that F103A is an N-terminal residue in the fusion peptide may also play a role in its ability to induce membrane fusion. Previous studies have demonstrated that altering the N-terminal residue of the influenza FP to various amino acid residues has different outcomes: either retaining complete fusion capability, losing complete fusion capability, or exclusively promoting hemifusion.^56,57^ This highlights the pivotal role of the N-terminal residue in the fusion process. The results presented here clearly indicate that having phenylalanine at the PIFP N-terminal position is critical for its fusogenic activity.

Overall, our findings shed light on the roles of the N-terminal phenylalanine and glutamine residues in the PIFP oligomerization, membrane perturbation, and leakage capabilities. This knowledge can be valuable for understanding viral fusion processes, as well as highlighting the significance of controlled fusion and leakage in the context of viral infection.

## Author contributions

M. V. and C. C. B. contributed equally to this work. M. V., M. N. M., C. M. S., and D. L. designed the computational systems and analysis and M. V. performed them. C. C. B., D. A. M., M. A. R. B. C., and A. S. V. designed the experimental assays and C. C. B. and D. A. M. performed the experiments. M. V. and C. C. B. wrote the manuscript with input from all authors. All authors have given approval to the final version of the manuscript.

## Data availability

The data supporting this article is available within the article and in a Zenodo Repository (https://doi.org/10.5281/zenodo.14967231). The raw files are available upon request.

## Conflicts of interest

The authors declare no competing financial interests or personal relationships that could have influenced the work reported in this paper.
